# EEPIC - Enhancing Employability through Positive Interventions for improving Career potential: the impact of a high support career guidance intervention on the wellbeing, hopefulness, self-efficacy and employability of the long-term unemployed - a study protocol for a randomised controlled trial

**DOI:** 10.1186/s13063-018-2485-y

**Published:** 2018-02-26

**Authors:** Nuala Whelan, Sinéad McGilloway, Mary P. Murphy, Colm McGuinness

**Affiliations:** 1 Centre for Mental Health and Community Research, Maynooth University Department of Psychology, National University of Ireland Maynooth, Co. Kildare, Ireland; 2 Ballymun Job Centre, Ballymun, Dublin 11 Ireland; 3 Maynooth University Department of Sociology, National University of Ireland Maynooth, Co. Kildare, Ireland; 4 Department of Business, Institute of Technology Blanchardstown, Dublin 15, Ireland

**Keywords:** Employability, High support career guidance, Positive psychological interventions, Long-term unemployed, Wellbeing, Labour market activation

## Abstract

**Background:**

Labour market policy (LMP) and its implementation have undergone rapid change internationally in the last three decades with a continued trend towards active LMP. In Ireland however, this shift has been more recent with ongoing reforms since 2012 and a concomitant move toward active labour market ‘work-first’ policy design (i.e. whereby unemployed people are compulsorily required to work in return for their social welfare benefits). Labour market policies vary from those that require this compulsory approach to those which enable the unemployed to move towards sustainable quality work in the labour market through upskilling (human capital approach). Despite this, however, long-term unemployment—a major cause of poverty and social exclusion—remains high, while current employment support approaches aimed at sustainable re-employment are, arguably, unevaluated and under examined. This study examines the effectiveness of a new high support career guidance intervention in terms of its impact on aspects of wellbeing, perceived employability and enhancing career sustainability.

**Method:**

The study involves a single-centre randomised, controlled, partially blinded trial. A total of 140 long-term unemployed job-seekers from a disadvantaged urban area will be randomly assigned to two groups: (1) an intervention group; and (2) a ‘service as usual’ group. Each group will be followed up immediately post intervention and six months later. The primary outcome is wellbeing at post intervention and at six-month follow-up. The secondary outcome is perceived employability, which includes a number of different facets including self-esteem, hopefulness, resilience and career self-efficacy.

**Discussion:**

The study aims to assess the changes in, for example, psychological wellbeing, career efficacy and hopefulness, that occur as a result of participation in a high support intervention vs routinely available support. The results will help to inform policy and practice by indicating whether a therapeutic approach to job-seeking support is more effective for long-term unemployed job-seekers than routinely available (and less therapeutic) support. The findings will also be important in understanding what works and for whom with regard to potentially undoing the negative psychological impacts of unemployment, building psychological capital and employability within the individual, and developing career trajectories leading to more sustainable employment.

**Trial registration:**

ISRCTN registry, ISRCTN16801028. Registered on 9 February 2016.

**Electronic supplementary material:**

The online version of this article (10.1186/s13063-018-2485-y) contains supplementary material, which is available to authorized users.

## Background

### Background and rationale

The recent global crisis and subsequent high levels of unemployment in many countries throughout the world have led to a greater focus on, and recognition of, the importance of labour market policy (LMP) and job-seeking [1]. In 2015, global unemployment stood at 197.1 million, a 27 million increase on the pre-crisis level of 2007 [2]. In fact, across countries and over time, levels of unemployment vary considerably, with current unemployment rates in the OECD as low as 3.1% in Japan (2016) and as high as 24.9% in Greece (2015), and with even higher rates recorded in the emerging and developing world [2]. In the case of Ireland, the unemployment rate over the last three decades has been described as a ‘roller-coaster ride’ culminating in a sharp rise of 15.1% in 2012, from a low of 4.4% in 2006, and a continuous decrease since, illustrating the variability within countries [3].

Thus, government reaction to fluctuating levels of unemployment is important in terms of supporting the unemployed, not only in helping them to re-access the labour market, but also to become resilient in times of high unemployment. Policy responses to unemployment are generally implemented through LMPs, which can differ across countries, but tend to encompass a variety of similar regulative measures that influence the interaction between labour supply and demand [2], while also addressing imbalances in, for instance, long-term unemployment, income support, skills shortages, discrimination towards ‘disadvantaged’ labour [4] and ultimately ensuring efficient labour market functioning [5]. These policies are important in that they are broadly designed to assist the unemployed and those facing barriers to employment to access the labour market.

At the same time, there is considerable epidemiological research suggesting that unemployment can have much deeper impacts than just the loss of manifest benefits of employment (i.e. financial remuneration), with evidence of impacts on both physical and mental health [6–9]. For example, many unemployed job-seekers experience decreased wellbeing [9], high levels of psychological stress [10], low self-esteem and job search self-efficacy [11], which can act as barriers to returning to work due to low levels of motivation and attendant ineffective job-seeking strategies [12]. Thus, many people who become unemployed are at increased risk of developing stress-related disorders or psychological distress which can distance them from the labour market and increase their likelihood of becoming long-term unemployed [13]. Nevertheless, interventions aimed at re-employment tend to concentrate on increasing human capital through work experience and skills training, subsidised and direct employment, and intensifying job search behaviour, with the expected outcome being improved labour market access. Given the compelling evidence for the negative impacts of unemployment on mental health and wellbeing, it is imperative that policy responses to labour market detachment include interventions that help alleviate these adverse impacts and maintain good mental health [7, 13–15].

LMPs which seek to support unemployed people are often defined as ‘active’ or ‘passive’; the latter focuses on income replacement and the welfare of the unemployed, without improving their labour market access. Active labour market policies, on the other hand, include labour market integration measures which aim to improve the employment prospects and wage outcomes for those who have difficulty accessing the labour market such as the unemployed or those threatened by unemployment. Increasingly, governments are using a so-called activation approach in LMP design, where benefit rules and employment or training services are shaped with a view to moving unemployed income benefit recipients into work [16]. In recent decades this approach has emerged in public policy design in North America, Australia and Western Europe [17]. Indeed, according to Martin (2014) [3], activation policies have become a buzzword in LMP with a global movement towards a more regulatory form of welfare whereby established welfare rights become more conditional on job-seeking efforts [18]. Nevertheless, despite its popularity, there remains ambiguity around activation in terms of what it means for policy and practice, with much of this uncertainty arising from how it has been implemented in various countries and under a variety of labels (i.e. workfare, work-first, labour market activation, welfare to work) [17].

This variation in activation policies across the developed world lies mainly in the intensity of their regulation. Some countries for example, the UK and the US implement a ‘work-first’ approach whereby the unemployed are required to work for their unemployment welfare. In contrast, countries such as Denmark and the Nordic states employ a ‘human capital’ approach which aims to enable access to more sustainable quality work in the labour market. Interestingly, job quality has been included in the OECD’s wellbeing framework and identified as a key component of individual wellbeing and a means to better economic performance. Having a job is crucial for our wellbeing, but the quality of that job and its impact on our lives is also important and has been found to be associated with both mental and physical health [7]. Research in Switzerland [19] found that using negative incentives in activation-focused LMP (ALMP) led to lower quality post-unemployment jobs, both in terms of job duration and level of earnings. Studies have also shown that work of poor psychosocial quality can have long-term health impacts [20] which can be significantly worse than long-term unemployment itself. A recent systematic review found that people’s perceptions of negative psychosocial factors in the workplace is related to their mental health [21], with harmful psychosocial job conditions such as low job security, low decision latitude, high psychological job demands and low co-worker support increasing the chance of mental health symptoms [22]. While activation has been shown to increase exits from unemployment, it is important that the aim of effective activation regimes should be to help people access quality jobs [3].

Relative to many OECD countries, Ireland has been slow to follow suit in terms of active LMP and activation in particular. Interestingly, the recent economic crisis (2008–2012), has driven a significant and unprecedented move in this direction. With the rapid rise in unemployment in the early years of the recession,^1^ the Irish government’s policy was proving insufficient in responding to the needs of job-seekers. For example, it was described as ‘under-examined, fragmented and lacking in ambition… passive and low intensity in character …’ (Sweeney 2011) [23]. In an attempt to contend with the overwhelming rise in unemployment, recent changes in LMP have prompted a shift from passive to active participation and the strengthening of conditionality with the unemployed now required to engage in job search and activation programmes in order to continue receiving social welfare support. This is comparable with the ‘work-first’ approaches in the UK, Germany, the US, Australia and other European countries, many of which have been developing their activation strategies since the early 1990s. There are particular similarities between the Irish model and UK welfare reforms principally in relation to the re-design of welfare services (i.e. Jobcentre in the UK and the Intreo service in Ireland), the implementation of conditionality [24] and the sub-contracting of re-employment services to private providers on the basis of performance-related results [3].

This shift towards activation was achieved through the implementation of the Irish Government’s LMP, ‘*Pathways to Work’* (Department of Social Protection [DSP], 2011, 2013, 2014, 2016–2020) [25], which has been precipitous, and despite an explicit focus on long-term unemployment, there is little evidence of targeted approaches which acknowledge long-term unemployment and/or its impact on psychological wellbeing. Although the policy refers throughout to prioritising and adequately supporting vulnerable groups including the young unemployed and long-term unemployed through the provision of activation services, the response in terms of application is increased frequency of engagement (i.e. one meeting with a case officer per month). Thus, while this new policy is widely considered to be a success in terms of reducing unemployment by the Irish Government [25] and in public discourse through the obvious decline in unemployment (15.1% in 2012 to 7.1%, Q4 2016), nothing is known about its impact on the wellbeing and sustainable re-employment of job-seekers in quality jobs and, in particular, the long-term unemployed. This is an important knowledge gap in view of the extensive literature linking unemployment to poor mental health and wellbeing [7, 26, 27], considerable evidence indicates that unemployed people are more likely to experience: anxiety, loss of confidence, low self-esteem, loss of motivation, suicidal ideation, low levels of coping, psychosomatic problems, poor cognitive performance, behavioural problems and paranoia [9].

While there is little evidence of the effectiveness of such programmes, there is much political interest in using ALMPs as a means of reducing levels of unemployment. One of the most cost-effective ALMP are ‘job search and assistance’ interventions which comprise measures aimed at improving job search efficiency such as job search courses, job clubs and intensified counselling [28]. Other components include monitoring and sanctions, which aim to incentivise job-seekers to actively seek work and exit the benefit system [29]. However, the effectiveness of ALMPs remains unclear, despite many experimental evaluations (e.g. randomised controlled trials [RCTs] and micro econometric impact evaluations); while these are a useful starting point, there is a need to examine programmes more closely in order to understand why they work for some and not for others [4].

Evaluations of ALMPs are mostly conducted using gold standard econometric impact evaluations and RCTs [4, 30, 31]. The effectiveness of these interventions is based on their impact on the re-employment of the job-seeker rather than the changes which take place within the individual (e.g. increased employability/improved wellbeing) that, in turn, enable and support re-employment. For instance, labour economists have provided evidence for the effectiveness of the various types of ALMPs available to job-seekers and how they might be used to reduce unemployment [28, 32]. This evidence suggests that some interventions can have a positive effect on re-employment. For example, Card et al. [33] found that job search assistance programmes were most likely to have positive impacts in the short term, with labour market training programmes impacting positively in the longer term. Interventions such as counselling and training were also found to increase transition rates for the unemployed into employment [34]. However, other findings are mixed where such interventions have been found to be unsuccessful or with little or no impact [4]. In one of the most influential meta-analyses of ALMP evaluations, Martin and Grubb [35] found that many ALMP programmes were ineffective or often counterproductive in assisting the unemployed to regain access to the labour market. For example, subsidised public sector employment programmes fared least well in terms of impact and improved access to the labour market [33]. Conversely, however, Kluve et al. found that there may be potential gains from matching participants and programme types, suggesting that programmes may work better for some than for others, depending on their labour market needs [36].

Current evidence [37] suggests that there is no ‘one-size-fits-all’ ALMP which can improve employability, but rather that a shift towards a more tailor-made or individualised approach in practice may be more effective. Interventions targeted at an individual’s needs, such as training and counselling, have been shown to have positive effects on wellbeing [38–40]. Similarly, evaluations of Cognitive Behavioural Therapy (CBT)-based employment programmes such as the ‘CHOICES for Well-being’ project [41] showed improvements in the mental health, self-esteem and job-search self-efficacy of participants, as well as a reduction in the occurrence of negative automatic thoughts and employment progression for some participants. Improvements also persisted at three-month follow-up. In a recent systematic review of interventions aimed at reducing the impact of unemployment on mental health, Moore et al. [15] reported that short one- to two-week job club-type interventions can reduce the risk of depression for up to two years, with the largest impacts seen in those who re-accessed the labour market. However, they found mixed evidence for CBT interventions, with only short-term effects on depression symptoms and re-employment in a trial with a longer (seven-week) CBT intervention [42] and no effects in a shorter (two-day) intervention [43]. The question of whether such interventions could be implemented to support the unemployed in overcoming the negative psychological impacts of unemployment remains unanswered. Moore et al. [15] conclude that more high-quality RCTs which follow established guidelines (e.g. CONSORT, SPIRIT) are needed to provide evidence of the effects on mental health, of interventions which could potentially be implemented to support the unemployed.

Psychologists and other social scientists have made important contributions towards understanding the impact of unemployment on an individual in terms of wellbeing [44], self-esteem [45] and the loss of the latent and manifest benefits of work [46]. However, very little is known about the effectiveness of activation as a policy approach, and the impact of ALMPs, in potentially undoing the negative psychological impacts of unemployment and building psychological capital and employability within the individual. Theories of employability, such as the model proposed by Fugate et al. [47], define employability as a person-centred psychosocial construct and something separate from the environment thereby providing the individual with the opportunity to identify their strengths and weaknesses in terms of personal factors [48]. This is particularly important given the rapidly changing labour market, with its lack of security and increasing demand for flexibility within the workforce.

In the case of the long-term unemployed, many have low or obsolete skills, which leaves them vulnerable to the risk of social exclusion and lifetime unemployment [37]. In addition, the negative impact of unemployment on psychological wellbeing has been found to worsen during the first year of unemployment [7]; thus, for job-seekers who have been out of the labour market for longer periods of time, the problems they encounter may overshadow their skills and abilities and can pose a significant barrier in terms of their ability to reconnect with the labour market [49]. Arguably, therefore, interventions designed for the long-term unemployed should aim to enable a change in the job-seeker’s career trajectory and assist them to access sustainable jobs rather than short-term precarious work where, after a few months, they may become unemployed once more. Yet the work-first approach assumes that any job is better than no job, reinforcing the sustainability of low-paid precarious work in the labour market [50].

Thus, it is important to investigate empirically whether long-term unemployed clients who receive needs-based individualised services become more employable by means of receiving a range of supports that focus on promoting greater self-awareness, improving wellbeing, increasing hopefulness for the future, and enhancing self-esteem and self-efficacy. For example, the most recent version of the Irish Pathways to Work 2016–2020 policy introduced a new strand called *Building Workforce Skills* which aims, through cooperation with the education and training sectors, to continuously develop the labour force and to provide job-seekers with the opportunities to develop the skills and competencies required to access and sustain employment.

As the Pathways to Work activation model is a recently established approach, no previous evaluations or comparable studies have been undertaken. However, a number of RCTs and pre–post comparisons have been conducted in other countries (e.g. Sweden [51], France [52], the UK [42] and the USA [53]) in order to assess the effectiveness of interventions on wellbeing and self-esteem in unemployed participants. These have included a variety of non-traditional employment-focused interventions including CBT, therapeutic training and individualised job search. However, there are few robust evaluations of *non-traditional* interventions targeted at individuals, their wellbeing and employability [4, 13, 15]. This provided the impetus for the present study.

### The current study: objectives

The principal aim of this study (called ‘EEPIC’) is to assess the impact of a newly developed therapeutic career guidance intervention—when compared to routinely available support—on the psychological wellbeing (including hopefulness and resilience) and perceived employability of a sample of long-term unemployed job-seekers in a disadvantaged urban setting. The goal of the intervention is to support the unemployed in strengthening their wellbeing, build hopefulness, resilience and career self-efficacy in order to improve employability, and increase access to sustainable labour market opportunities.

This new high support intervention uses a career/vocational guidance approach and aims to increase levels of psychological wellbeing when compared to current employment support services (Pathways to Work) provided to the long-term unemployed. In terms of ALMPs, the intervention could be categorised within the OCED’s classification as a ‘Job Search Assistance’ programme. A full description of the intervention vs usual services is provided in Table 1. This new high support intervention is designed to: (1) increase levels of wellbeing in the long-term unemployed; and (2) help to improve their employability.

**Table 1:** Aspects of service as usual vs intervention

Aspects of service	Service as usual	Intervention
Profile form detailing individual needs and barriers to progression		x
Tailored career guidance process		x
Career plan – with short- and long-term goals (agreed after the guidance process)		x
Stated importance of relationship building between client and practitioner		x
Personal progression plan (agreed at 1st meeting)	x	
Implementation of career plan with support of guidance practitioner		x
Review meetings	x	x
Timing of meetings	Indicated by PEX profiling score	Indicated by need as identified by practitioner/client
Number of meetings	3–4 over 6-month period	3–6 over 6-month period

### Trial design

**Fig. 1:**
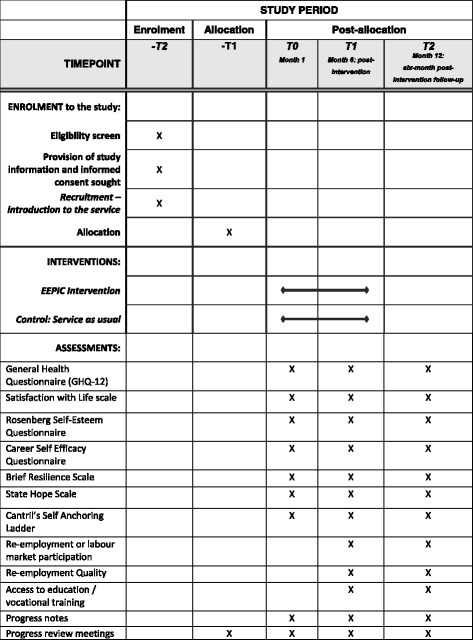
SPIRIT figure: EEPIC enrolment, intervention, and assessment

The EEPIC study is a single-centre, partially blinded RCT, with two parallel groups and a primary outcome of wellbeing and a secondary outcome of perceived employability, at post intervention and at six-month follow-up. The principal hypothesis is that participants receiving the high-support intervention will have significantly better wellbeing and employability outcomes post intervention and at six-month follow-up, when compared with participants receiving services as usual. The trial has been designed in accordance with the SPIRIT (Standard Protocol Items: Recommendations for Interventional Trials) Statement and Checklist (see Additional file 1) and CONSORT (Consolidated Standards of Reporting Trials) criteria [54–56]. For more information on the trial schedule, see the SPIRIT figure (Fig. 1).

## Methods: participants, interventions and outcomes

### Study setting

The EEPIC study is being implemented in a non-governmental organisation (NGO) contracted by the DSP in Ireland to deliver public employment services locally to the unemployed. The NGO is situated within an urban area characterised by socioeconomic disadvantage and which has been classified as ‘Very Disadvantaged’ by the All-Island HP Deprivation Index (2011). This classification is based on demographic profile, social class composition and labour market situation [57]. The unemployment rate for the area has remained consistently high since the 1980s and is approximately three times the national average, standing at circa 31% (based on CSO data, September 2015 [58]).

### Participants and eligibility criteria

Participants in this study are unemployed male and female adults aged 18–60 years who are in receipt of a job-seekers payment for a minimum of 12 months. In Ireland, unemployed people are paid either a Job Seeker’s Allowance (JSA) or a Job Seeker’s Benefit (JSB) weekly through the Department of Social Protection. JSB is paid for nine months and its recipients are people covered by social insurance (PRSI). When a person reaches the end of the nine-month period, or if they do not have enough PRSI contributions, they may be entitled to a JSA which is a means-tested payment. The majority of participants in this study will be in receipt of JSA in order to meet the 12-month unemployment criterion for entry into the trial. Some participants, however, will be in receipt of a Job Seeker Transition payment which is available to lone parents whose youngest child is aged 7–13 years.

Study participants are clients of the DSP’s public employment service called *Intreo* which offers clients a single point of contact for all employment and income supports. Participants are referred by the Intreo office to Pathways to Work (Activation) and will have attended a Group Information Session (GIS) in the Intreo service. Participants are recruited thereafter and before starting a job assistance intervention. Exclusion criteria are evidence of a serious mental health problem and/or drug misuse. Participants who do not attend their first post-GIS appointment following at least three attempts to engage them and who have been referred back to Intreo are also excluded from the study. Participants must provide written informed consent before taking part in the study.

### Eligibility criteria for staff delivering the interventions

Staff delivering the new intervention have been selected on the basis of their experience of working in a high-support way on similar interventions such as the *Emerge Mount Street Employment*^2^ initiative and the *Ballymun Youth Guarantee*^3^ pilot. Staff must also have relevant training and skills in the use of key guidance approaches and tools (e.g. interest inventories, vocational counselling skills, motivational interviewing).

## Interventions

### The EEPIC intervention

**Fig. 2:**
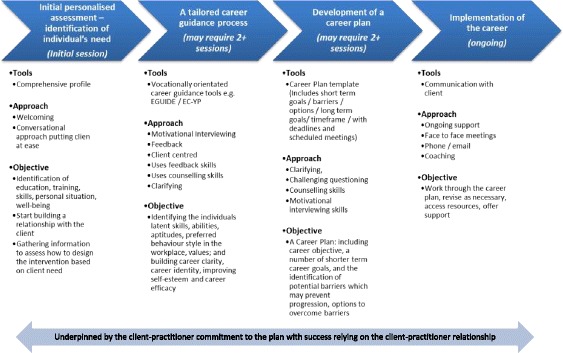
Four Stage EEPIC Intervention Process

The new EEPIC intervention is a high-support therapeutic guidance programme which focuses on the development of a career plan and strengthening the human, social and psychological capital required to implement this plan**.** The intervention consists of a four-stage process (see Fig. 2), which typically lasts 8–12 weeks, and which aims to support the job-seeker in developing the skills necessary for labour market access while building self-efficacy and esteem and improving psychological wellbeing:

- *Stage 1:* The individual’s needs (education, training, skills, personal situation, employment history, perceived employability competencies, work values, barriers to employment, wellbeing, etc.) are assessed using a Profile Form adapted from the Ballymun Youth Guarantee (Ballymun Job Centre, 2013) and EMERGE (Ballymun Job Centre, 2010–2012) initiatives. Identification of specific needs and their severity is vital in understanding the barriers faced by the individual and the types of supports and actions required to enable them to move towards the labour market. The outcome of the individual needs assessment determines the extent to which guidance practitioners may need to support the individual to engage with appropriate services to address issues which pose barriers to progression (e.g. addiction, literacy). Interaction with other services and supports are documented by the practitioner in their case notes.
- *Stage 2:* A tailored career guidance process is implemented to support the job-seeker in identifying latent skills, abilities, aptitudes, preferred behaviour style in the workplace and values. This process aims to build career clarity, career identity and improve self-esteem and career efficacy. Vocationally orientated career guidance tools and approaches (e.g. career interest inventories, general and specific aptitude assessments, person-centred vocational counselling) are used to reveal hidden strengths, aptitudes and preferences, while limitations are also acknowledged and documented. This information is used to inform the development of a detailed career plan.
- *Stage 3:* The job-seeker and guidance practitioner work together to develop a career plan which includes a career objective or aspiration, a number of shorter-term career goals which should be SMART (Specific, Measurable, Achievable, Realistic and Time-bound) and potential barriers to progression. A timescale for this plan is also identified and a method to achieve it is discussed, particularly in relation to responsibilities and extent of contact required (e.g. weekly/fortnightly meetings with the guidance practitioner).
- *Stage 4:* The career plan is implemented in a supportive and positive way. This involves the job-seeker and the practitioner working together to accomplish the planned career goals, to maintain levels of motivation, to build resilience against setbacks and adapt and re-plan as required.

This intervention is implemented on a one-to-one basis with the guidance practitioner and the client working together to identify key strengths, career identity and learning needs. The successful implementation of a career plan relies heavily on the client–practitioner relationship and commitment to the plan. This intervention is, therefore, highly dependent on the skills and approach of the practitioner involved in delivering the service. It also relies on the continuum of support offered so that the client is supported throughout their journey toward, and into, the labour market. This involves building networks with those who can offer support, such as mentors within the education and training sector and within the workplace.

### Adherence to intervention protocol

Face-to-face adherence meetings will be held with practitioners to monitor participant progress and their adherence to the intervention. These meetings will form part of the already established guidance sessions which are held bi-monthly and are attended by the guidance team leader and the guidance practitioner with the purpose of reviewing progress. The lead investigator (psychologist) will attend these meetings and monitor adherence to the intervention and study protocol.

Participants will be permitted to attend additional support services with which they are in contact before trial entry or identified as appropriate to their needs during the trial. These support services include primary healthcare, addiction supports and community services, but exclude other employment support services. Participants who are referred by the DSP to an alternative employment support service either before their entry to the study or who are referred during the trial will be ineligible for participation.

### Control group: ‘service as usual’

Control group participants receive the ‘service as usual’ as provided nationally by the DSP’s Intreo service, the Irish state public employment service. This service is also delivered within the NGO and consists of a number of steps:

- *Step 1:* Once the individual has attended a GIS, a first appointment is made, the timing of which is determined by the individual’s score on a statistical profiling model, ‘PEX’, which can be classified as ‘low’, ‘medium’ or ‘high’. The ‘Probability of Exit’ or ‘PEX’ profile, introduced in October 2012, is based on a number of factors including: history of long-term unemployment; age; number of children; level of education; literacy/numeracy issues; urban living; transport availability; levels of labour market engagement; spousal earnings; and geographic location. All of these can affect a person’s probability of remaining unemployed for 12 months or more and therefore becoming classified as ‘long-term unemployed’ [59]. Clients who have a low probability of exiting the live register within the coming 12 months receive more frequent interaction with the employment services than those classified as having a high probability of leaving the live register and accessing the labour market.
- ‘High PEX’ clients are invited to attend a meeting with a case officer six months after attendance at the GIS.
- ‘Medium PEX’ clients attend within two weeks.
- ‘Low PEX’ clients attend immediately.

At this first appointment, the client and case officer agree a number of steps or goals which the client commits to undertake as part of a Personal Progression Plan (PPP). This plan is signed and becomes the client’s responsibility to fulfil. Within the current study, case officers are also required to use the *Cantril’s Ladder* scale at the first appointment to assess the client’s perceived progress towards the labour market.

- *Step 2:* Case officers decide on and conduct systematic follow-ups (e.g. phone call, email, text) after the first meeting in order to ‘check in’ with the client and to see how they are progressing. The level of contact is normally agreed in the PPP and a follow-up category is set in the Client Services System (i.e. the DSP’s IT database) which calculates when the client is due for systematic follow-up.
- *Step 3:* The case officers are required to conduct Activation Review Meetings (ARM) by the DSP which can include a phone call or a face-to-face meeting to review progress of the tasks identified and agreed in the PPP. This is essentially a monitoring meeting and the timing of these meetings is dependent on the client’s initial PEX score:
- ‘High PEX’ clients receive an ARM meeting at six months and every three months thereafter;
- ‘Medium PEX’ clients receive an ARM meeting every three months;
- ‘Low PEX’ clients receive an ARM meeting every two months;
- Under 25 s (High, Medium and Low PEX) receive monthly ARM meetings.

Within the current study, case officers will also be required to use Cantril’s Ladder at the ARM meeting to assess perceived progress towards the labour market.

## Outcome measures

### Primary outcome measures

Overall psychological wellbeing will be assessed using two measures, the *General Health Questionnaire* (GHQ-12) and the *Satisfaction with Life scale* (Table 2). The *GHQ-12* is a 12-item self-report questionnaire most widely used to assess levels of psychological distress and to screen for minor psychological disorders [60]. The GHQ has been widely validated and shown to be highly reliable, with a reported Cronbach’s *a* in the range of 0.82–0.90 [61].

**Table 2:** Primary and secondary outcomes and data collection

Outcomes	Method of collection	Assessment	Baseline (t0)	Post Intervention (t1)	6-month follow-up (t2)
Primary outcome	Increased wellbeing	General Health Questionnaire (GHQ-12)	x	x	x
		Satisfaction with Life scale	x	x	x
Secondary outcomes	Self-esteem	Rosenberg Self-Esteem Questionnaire	x	x	x
	Career self-efficacy	Career Self Efficacy Questionnaire	x	x	x
	Resilience	Brief Resilience Scale	x	x	x
	Hopefulness	State Hope Scale	x	x	x
	Perceived progress towards the labour market^a^	Cantril’s Self-Anchoring Ladder^a^			x
	Re-employment or labour market participation			x	x
	Re-employment quality	Job satisfaction		x	x
		Job sustainability		x	x
		Level of earnings		x	x
	Access to education / vocational training			x	x

^a^Perceived progress towards the labour market is collected by the guidance practitioner during the intervention/ usual service, at a minimum of two time points, i.e. first appointment and last appointment

The *Satisfaction with Life scale* is a five-item self-report questionnaire developed to measure global cognitive judgemental aspects of life satisfaction [62]. Life satisfaction has been identified as the cognitive judgemental component of subjective wellbeing where judgements of satisfaction are dependent on a comparison with a person’s own standard as opposed to a criterion set within the scale or in a particular domain [62].

### Secondary outcome measures

Data will be collected for eight secondary outcomes (Table 2) which have been shown to benefit the unemployed in terms of mental health and increased employability. *Self-esteem* will be measured by the *Rosenberg Self- Esteem Questionnaire* [63], a ten-item scale designed to measure global self-esteem. *Career self-efficacy* will be measured by the *Career Self-Efficacy Questionnaire* which was adapted by Kossek et al. [64] from Sherer and Adam’s [65] General Self-Efficacy Scale to measure a context-specific form of self-efficacy. This is an 11-item self-report questionnaire which measures an individual’s belief in his or her ability to manage their own career.

*Resilience* will be measured by the *Brief Resilience Scale* [66], a six-item self-report questionnaire designed to assess the ability to bounce back or recover from stress. *Hopefulness* will be assessed using the *State Hope Scale*, a six-item self-report scale which examines goal directed thinking in a given moment [67]. *Perceived progress towards the labour market* will be measured by *Cantril’s Self-Anchoring Ladder* [68], a ten-step ladder where the top of the ladder represents the best possible situation for an individual and the bottom of the ladder represents the worst possible situation. The scale has been used in research as a type of wellbeing assessment and measures wellbeing as defined by judgements of life or life evaluation [69]. However, this scale has been adapted for the current study so that the focus is on career goals and the best and worst possible situation for the individual in relation to their career.

*Re-employment or labour market participation* will be assessed by rates of progression into employment post intervention (T1) and at six-month follow-up (T2). This will be measured by a single item which asks individuals to indicate whether they are ‘currently unemployed’ or ‘currently employed’. The *quality of re-employment* will be assessed in terms of:

- *Job satisfaction:* single item answered on a 4-point scale (‘All in all, how satisfied would you say you are with your new job?’) [70].
- *Job sustainability:* single item answered on a 7-point scale (‘How likely is it that you will actively look for another job in the next year?’) [71].
- Satisfaction with *level of earnings* will be rated on a 5-point scale ranging from ‘very dissatisfied’ to ‘very satisfied’.

*Access to education/vocational training* will be assessed by rates of progression into education and /or training and its relevance to the individual’s career plan post intervention at T1 and at T2. This will be measured by a single item which asks individuals to indicate whether they have completed an education or training course relevant to their career plan, are currently registered on an education or training course relevant to their career plan, are waiting to start an education or training course relevant to their career plan or are not participating in education or training.

### Participant timeline

The scheduling of study phases is outlined in Fig. 1 with the overall study anticipated to run for a period of 24 months. This timeframe ensures that participants have sufficient time to receive an individualised service and to participate in a six-month post-intervention follow-up in order to ascertain to what extent any changes from either intervention are maintained, improved or have deteriorated over time. Enrolment into the study is on a phased basis and is dependent on the referral of job-seekers to activation and the GIS. Additionally, both the intervention and control group participation durations may vary per job-seeker, due to the individualised nature of the services, but will not exceed six months.

### Sample size

A power analysis was conducted using the primary outcome measure of overall psychological wellbeing (GHQ-12) in order to identify the minimum sample size required to detect an increase in wellbeing post intervention. As already indicated, the Pathways to Work activation model is a recently implemented approach and so no previous evaluations or comparable studies have been undertaken, although similar studies had been conducted in Sweden [51], France [52], Australia [6] the UK [41, 42] Finland [72] and the USA [53, 73]. Analysis of these studies indicate varying sample sizes (*n* = 16–1200); thus, to ensure the minimum sample size is achieved and the study is powerful enough to detect significant differences between the groups, a power analysis was performed to establish a realistic estimate based on the primary outcome measure. This analysis was conducted for an independent samples t-test, as this is expected to be the least powerful test in the overall main analysis. The analysis was two-tailed (alpha of 0.05), as we do not know in advance which group will perform better in GHQ-12 terms. G*Power t-test calculations for the difference between two independent means (two groups) show that for the current study, 128 unemployed participants (64 in each group) will be sufficient to detect a change of 0.50 (medium) at 80% power and at 5% significance at a given time point. An allowance of (approximately) 10% will be made for possible attrition, so the actual sample size target will be 70 per group. This should be more than sufficient for Mixed Model Repeated Measures (MMRM) across the three time points to have > 80% power and also allow for post-hoc t-tests both between groups and within groups to have ≥ 80% power.

### Recruitment

Participants in this study will be randomly selected from a pool of job-seekers, referred by the Intreo office to the NGO for activation (i.e. service as usual) on a weekly basis. Referred job-seekers consist of a mix of short- and long-term unemployed. The job-seeker is invited to attend the first step in the activation process which comprises a GIS where information on all supports and interventions offered by the public employment service and delivered through the DSP’s Intreo service, are outlined. The GIS normally occurs within two weeks of a social welfare claim being made; however, due to the large number of job-seekers in Ireland who are currently long-term unemployed (100,600 individuals accounting for 56.1% of total unemployment [CSO, Q1 2016]), an accumulation of job-seekers in the Intreo system has resulted in job-seekers who have not yet attended a GIS. In response to this, the Intreo office identifies a specified number of long-term unemployed job-seekers each week for referral to the NGO employment services. Currently, 60 job-seekers per week, with varying durations of unemployment, are drawn randomly from the live register for attendance at a GIS which is held in the NGO and delivered by a NGO staff member. The GIS is a standard presentation, designed by the DSP, and delivered nationwide to all job-seekers as part of their initial engagement with the employment services.

The list of job-seekers referred to the GIS is sent to the NGO data manager one week before the GIS. Clients are allocated to one of seven guidance practitioners and appointments made on the NGO appointments schedule. Clients attend the GIS and are informed of their appointment (i.e. given an appointment card with time and name of guidance practitioner for the following week). Client’s first appointments are with the researcher who at this point invites eligible clients to participate in the study and written informed consent is sought. This process will continue until adequate participant enrolment has been achieved for each group.

## Methods: assignment of interventions

### Allocation

#### Sequence generation and implementation

The participant’s initial appointment is with the researcher who explains the study and consent forms and administers the participant questionnaire. The researcher informs the data manager of those clients who agree to participate and who give informed consent. The data manager randomly assigns eligible participants to either the intervention or control group on a 1:1 basis using the SNOSE (sequentially numbered opaque sealed envelopes) method as described by Doig and Simpson [74]. Randomisation is conducted by the data manager only, ensuring that the randomisation is achieved without any influence from the researcher or the practitioners involved in the delivery of the service.

Seven practitioners deliver services to the intervention and control groups. Practitioners who deliver the intervention are not involved in delivering services to the control group (and vice versa) in order to ensure that practitioners deliver the service with fidelity and that there is no contamination between the intervention and control groups.

#### Allocation concealment mechanism and implementation

The data manager has been provided with 200 sealed envelopes containing a treatment allocation paper with either ‘Intervention A’ or ‘Intervention B’ (control group) printed on one side. Using the SNOSE (sequentially numbered opaque sealed envelopes) method as described by Doig and Simpson [74], the data manager allocates participants to either the Intervention or Control groups. Participants are also tagged on the NGO’s internal data management system as Intervention or Control group so that reports can be accessed at required junctures in the study. The data manager informs the researcher of any issues arising, such as a delay in referrals from Intreo, a break in the referral cycle or issues relating to randomisation.

### Blinding

After assignment to the respective interventions, the participants are blinded to allocation for the duration of the study. The researcher who performs the assessments at baseline is also blinded until the completion of baseline assessments. Due to the nature of the intervention, the staff delivering the interventions cannot be blinded and are instructed not to disclose information to either the researchers or the participants which may indicate which intervention the participant is receiving. Blinding of the researcher cannot be maintained post baseline as the researcher is also responsible for data collection in this study. For the participant, it remains unclear which intervention is being received as both interventions occur within the same site. For the researcher, knowledge of the staff responsible for each intervention and their caseloads indicates which intervention the client is receiving and therefore the blind is broken once the intervention starts.

The conditions under which the blind can be broken are limited to exceptional circumstances where the practitioner working with the client identifies the need to reveal the intervention. Such cases include situations where the allocated intervention is deemed harmful to the individual, particularly with regard to ancillary services, such as medical care, mental health care or social care. Practitioners will be required to discuss such cases in the first instance with the lead investigator (who is also an employment practitioner).

### Methods: data collection, management and analysis

#### Data collection methods

Participants are invited, as part of the trial, to complete a range of assessments at several time points (i.e. at baseline, at post intervention / ‘service as usual’ and six-month post-intervention follow-up) in order to measure the impact of the intervention or ‘service as usual’ on key dimensions including self-esteem, hopefulness, resilience and career self-efficacy (Fig. 1).

Each questionnaire (see Additional file 2) is coded with client ID, date of completion, researcher’s name, and questionnaire version (i.e. baseline, T1, T2). Client IDs are generated by the NGO and link to the client’s personal information contained on the NGO’s client database. This will be beneficial to the researcher at the six-month post-intervention phase in order to update data on the intervention or ‘service as usual’ and outcomes.

At baseline (T0), the study is explained to the potential participant and consent is sought (see Additional file 3). The baseline questionnaire is administered, coded and signed by the researcher. At post intervention (T1), the researcher meets with each participant, administers the questionnaire along with a participant update questionnaire which aims to capture information on re-employment, quality of employment, training progression and overall progress (see Additional file 4). This process is repeated at the six-month follow-up (T2).

A tracking file containing participant details—including client ID, completion of questionnaires, appointment dates, guidance practitioner name and outcome updates—will be maintained by the researcher for the duration of the study. Due to the nature of this client group, non-attendance is common and so a tracking system enables the researcher to identify ‘no-shows’, ‘drop outs’ and patterns of attendance. Outcome data for participants who do not continue or who deviate from the intervention or ‘service as usual’ will be documented in this file to study completion.

Practitioners to whom intervention clients are referred, are required to complete an in-depth profile for each participant relating to education, previous employment, skills, values, perceived employability and barriers to progression. These data will be held by the practitioner until the intervention is complete. All practitioners are required to administer the ‘P*erceived progress towards the labour market’* measure, Cantril’s Ladder, at baseline and post intervention, although some practitioners may choose to administer this on a more regular basis.

A small token in the form of a voucher is offered to each participant to help increase participation and to thank participants for their time in completing questionnaires at post intervention and at six-month follow-up.

#### Data management

Data management will be overseen by the researcher who will implement checks on a monthly basis to ensure the quality of the data collected and the accuracy of electronic data entry and coding. The researcher will gather all questionnaires completed at each time point and ensure the correct coding has been used and the appropriate date is on the front cover. This information will be entered into a database (IBM SPSS statistics version 22) on the researcher’s encrypted laptop and backed up every week on a separate removable storage device (also encrypted) which is stored safely in the researcher’s office. Data collected by practitioners will be gathered by the researcher post intervention and entered into the SPSS database for analysis. The tracking file will be updated by the researcher to ensure the visibility of each participant’s engagement with the service and their participation in the trial. All hard copies of questionnaires will be held securely in a locked cabinet for ten years after completion of the study, after which they will be destroyed. Participant identifiers will be stored separate from the data. The coding key and electronic raw data will be held securely for ten years and will then be destroyed by the researcher.

#### Statistical methods/analysis

The null hypothesis states that there will be no difference between the two groups in terms of primary outcomes (wellbeing) and secondary outcomes (self-esteem, career self-efficacy, resilience, hopefulness, perceived progress toward the labour market) at post intervention and at six-month follow-up. Descriptive statistics will be used to describe the pre-treatment characteristics of participants. Baseline analysis will be conducted to establish the internal consistency of the outcome measure scales, where a Cronbach’s alpha of above 0.7 will be required. Previous studies have reported Cronbach’s alphas of at least 0.7 across all measures*.*

The study will use a randomisation technique (the SNOSE [sequentially numbered opaque sealed envelopes] method as described by Doig and Simpson [74]) which ensures that participants from both groups come from the same population. Pre-treatment analysis will be conducted on primary and secondary outcome measures to show, for example, levels of wellbeing (primary outcome) as indicated by GHQ-12 scores in comparison with appropriate established norms *(e.g. national wellbeing data, HRB (2008))* so as to indicate how job-seekers present for activation services.

Mixed Model Repeated Measures (MMRM) will be used to investigate the effects of the intervention on primary and secondary outcome measures (i.e. wellbeing and employability). Continuous outcome data, including the primary outcome measure of wellbeing and five of the eight secondary outcomes which have been shown to contribute to mental health and increased employability (i.e. resilience, career-efficacy, hopefulness, self-esteem, perceived progress towards the labour market) will be analysed using MMRM. Where parametric test assumptions fail significantly, then non-parametric tests will be used.

MMRM will be used to investigate effects at two between (intervention and control) and three within (pre-intervention, post-intervention and six-month follow-up) levels. Initial MMRM analysis will control for age as a fixed co-variate, along with gender, and duration of unemployment or highest educational level, as applicable. Modelling for the primary outcome will be conducted using an unstructured repeated measures co-variance matrix and all other variables as fixed effects.

MMRM was chosen as the main statistical method for analysis as it can reduce several analytic problems that may arise from the EEPIC study design. First, it has the advantage of modelling change within individuals as well as across groups, thus enabling the isolation of factors contributing to the outcome, such as, age, gender, duration of unemployment or highest educational level (common to both intervention and control condition). Second, it allows for different numbers of measurements per participant, thereby tolerating a level of missing data, which are a particular problem with RCTs as follow-up data are often collected many months after treatment has ended and participants may be difficult to contact [75]. This enables us to use all of the data collected as opposed to deleting cases or imputing missing values. Third, it has the advantage of allowing for different time points for each individual, so data collected for one participant at month 4 can be tested alongside data collected for the next participant at month 6 [76]. Singer and Willet [77] identify this as the best approach for longitudinal data which has three or more time points.

The analysis will follow an intention-to-treat (ITT) principle where all randomised participants, including those who stop receiving the intervention, will be analysed ‘as randomised’. MMRM analysis is a maximum likelihood statistical modelling technique whereby mean estimates and the repeated measures covariance structure for the observed data are based on a statistical model and possible values are generated for the missing data [78]. Attrition will also be analysed to assess the differences between those who ‘dropped out’ and those who stayed, and indeed if there are predictors at baseline to indicate same. MMRM will be used in the main, although t-tests will be employed to detail any significant differences found from the MMRM.

In addition, descriptive statistical summaries (means, standard deviations, frequencies) will be presented for primary and secondary outcome measures at each time point (baseline, post intervention and six-month post intervention). Of most interest will be the identification of changes in primary and secondary outcome measures at group level between T0 (baseline) and T1, and T0 and T2, and between T1 and T2. Additional descriptive analysis (e.g. frequencies) of the re-employment (secondary) measure will be conducted to assess the differences between the two groups in terms of their re-employment outcomes.

Sub-group MMRM analysis will be conducted to investigate if the intervention effects differ for certain participant groups, based on variables such as gender, age, education level and unemployment duration. T-tests and Chi-squared tests will be employed to identify mean differences and associations with regard to primary and secondary outcome measures.

A full statistical analysis plan (SAP)—in the form of a Trials (free) update—will be provided once all data are gathered and before opening the database. Analysis will be conducted using SPSS software (IBM SPSS Statistics version 22).

### Methods: monitoring

#### Data monitoring

A data monitoring committee is not feasible for this trial due to its short duration and size. The researcher will have sole access to the data and will monitor it monthly to ensure that the quality of data is maintained throughout the trial. Furthermore, within the context of this trial, an interim analysis is not practicable as sufficient data may not be available at the interim point for analysis. However, the researcher through monitoring of the data will inform the NGO, should any issues arise with the data collection, the recruitment of participants or the implementation of the intervention. This is of particular importance due to the ongoing changes in LMP implementation in Ireland and its very real bearing on the trial progress. Nevertheless, the flexibility of the NGO will ensure that should any changes to the trial be required, they will within reason be facilitated.

#### Harms

There are some (minimal) risks envisaged in this study. From the researcher’s experience of working with job-seekers, there can be a tendency for the client to disclose personal information that may not be sought within the interview/focus group and to express their own experiences, difficulties and barriers and expect that the researcher may be able to offer further assistance. In practice, this involves setting and recognising clear boundaries while still providing an open and supportive environment within which the participant can engage in the interview/questionnaire completion.

Completion of the GHQ-12 may cause some minor distress, but the researcher is an experienced administrator of this measure and other similar questionnaires, as well as having well developed test administration skills. Close adherence to the British Psychological Society Code of Good Practice for Psychological Testing and the Psychological Society of Ireland Code of Ethics will also ensure that any risk will be managed according to best practice. If the client has a negative reaction to the administration of the questionnaires, a referral to an experienced guidance officer (i.e. the client’s case worker) in the DSP/NGO and the primary healthcare team will be made. In addition, information on a range of support services will be given to the client.

Other potential risks will be addressed by ensuring that there is appropriate local information pertaining to support services available. Such services include counselling services, addiction services, Local Employment Centre services and other community-based services. The researcher’s own training as a psychologist and experience of working with numerous disadvantaged clients will also ensure that each participant is treated with respect and that any signs of distress will be appropriately identified and the participant referred immediately to a suitable service(s).

Questionnaires will be administered in the NGO, which has, through its own Health and Safety policy, procedures in place regarding the safety of clients and staff. These procedures will be followed alongside the National University of Ireland Maynooth, Department of Psychology guidelines ‘Guidance for safe working practice in psychological research’.

Further to the protection afforded by the above policies and guidelines, participants will be provided with a detailed and easily comprehensible information sheet and an informed consent sheet (see Additional file 3) and will be reminded of their option to withdraw from the study at any time (up until the point of data analysis) should they so desire.

#### Auditing

Auditing will not be necessary in this study due to its short duration.

### Ethics and dissemination

#### Protocol amendments

Should any amendments to the protocol be required, particularly those which may impact the trial and its implementation or the participants and their outcomes, a formal amendment to the protocol will be required. This will necessitate approval from the funder, the NGO and the National University of Ireland Maynooth Social Research Ethics Committee. Administrative amendments which do not impact on the trial and participants will not require formal approval, but will be documented by the researcher in the tracking file.

#### Consent or assent

Consent is sought from participants involved in the study at the first meeting with the researcher. Each participant is provided with an information sheet (Additional file 3) outlining the background to the study, the rationale and the objectives. Participants also receive a consent form (Additional file 3) which they are asked to sign; a copy is given to them to retain for their own records. The researcher also talks through both documents to ensure they are properly understood by the participants. Verbal consent will be sought if any issues regarding poor literacy arise.

All participants in this study who may be considered potentially vulnerable are in receipt of a Job Seekers payment, thereby deeming them fit for employment. It is likely, therefore, that participants are capable of consenting to participation. However, assent is also sought on occasions where the researcher has concerns regarding the participant’s understanding of the process. The researcher also talks through both the information sheet and the consent forms to ensure they are properly understood by the participant.

As this study requires participation on more than one occasion, participants will be contacted before the follow-up assessment (post intervention and six-month follow-up) and continued consent will be sought before the follow-up study commences. Again, a copy of the consent form will be given to participants as soon as possible after consent has been obtained. If the participant does not wish to continue, they may withdraw at any time. Completion of the withdrawal slip which forms part of the information sheet will be requested for the researcher’s records. Data can/will be withdrawn up until the point of completion of data entry. Consent and continued consent are sought solely for this study as no ancillary studies are planned.

Participants are informed in the information sheet of the ongoing nature of this study and will be informed throughout of their right to withdraw participation up until the point of data entry without penalty. As this study is closely linked to the services provided by the Department of Social Protection, participants may have concerns that non-participation may have a negative effect on their social welfare payment. The information sheet and the informed consent form clearly indicate that there is no conditionality related to this study and that no penalties apply for non-participation. Furthermore, participants are informed that they may, at any time, contact the researcher should they have concerns regarding their participation. Participants are also informed by email/post when each aspect of the study relating to their participation is complete and may request a summary of the research findings when it becomes available.

#### Confidentiality

All identifying information is removed from the data in order to protect the safety and integrity of the research participants. Each participant is allocated a unique identifier at the point of consent and is informed of this in the consent form. A document (encrypted and password-protected) containing the coding key is only accessible by the researcher and is located on a removable storage device in a locked filing cabinet in the researcher’s office.

All coded data are stored on the researcher’s computer and protected by encryption software (McAfee Endpoint Encryption), and backed up every week on a separate removable storage device (also encrypted) which is stored safely in the researcher’s office. The coding key and electronic raw data will be held securely for a minimum of ten years after completion of the study, after which they will be destroyed.

In addition, the information sheet alludes to the fact that: (1) this study will be published and the key findings presented at conferences and other public fora; (2) that all identifying information will be removed at the point of consent; and (3) that nobody will be identified in any publications.

Participants are also made aware that there may be instances where the researcher cannot maintain confidentiality, for example, where participant’s safety or wellbeing, or indeed the safety of others is at risk, and that a referral to the relevant services (e.g. mental health service) may be required.

### Declaration of interests

The authors declare no competing interests. The NGO research site is funded by the Department of Social Protection and is therefore contracted to deliver employment services which are subject to change dependant on current government LMP.

#### Access to data

The researcher, authors and the NGO will be given access to the cleaned dataset at the end of the study. The dataset will be password-protected and will be housed on a server in the NGO. The anonymised data will be made publicly available, as required by registration with the ISRCTN and upon request to the NGO.

#### Ancillary and post-trial care

Participants will be provided with post-trial care in the form of referral to ancillary services, such as primary healthcare, including mental health, counselling services, addiction services, local employment services and other appropriate community-based services, should they be required. The researcher and practitioners implementing the intervention or usual service will monitor participants’ responses to the services, and in the unlikely event that concern for a participant arises, particularly in terms of negative or adverse impacts stemming from their participation in the trial, a referral will be made immediately to a suitable service(s).

Should this study provide evidence of the effectiveness of the EEPIC intervention in improving wellbeing and employability, participants who do not receive the intervention (but who receive the PTWP service) may receive the intervention at a later point if agreed by the DSP. The researcher will make a strong recommendation to the NGO and to the DSP, that those who participated in the control group be offered this service as soon as possible.

#### Dissemination policy

Trial results will be disseminated to participants, employment services, relevant government departments and other interested organisations (e.g. charities, social justice organisations, community-based services). Findings will also be presented at appropriate academic conferences and seminars and published in peer-reviewed journals and on relevant websites (e.g. the NGO website). As indicated above, the trial has been registered with ISRCTN, and has been promoted at community level, and with wider employment services and the DSP. A summary report of the findings will be prepared for the NGO and recommendations made for policy and practice. In addition, a number of academic manuscripts are anticipated: (1) a paper on the major outcomes from the study; and (2) a paper detailing specific aspects of the study. The authors of these papers will be those listed as the protocol contributors. Anonymised data will be made publicly available through the Irish Social Sciences Data Archive (ISSDA) and the Irish Qualitative Data Archive (IQDA) as required by registration with the ISRCTN. This will be available within six months of the trial end date.

## Discussion

The current trial is the first of its kind in Ireland and one of few internationally to examine whether interventions which aim to build employability by targeting individual wellbeing are more effective than conventional ALMPs and activation approaches. The EEPIC trial is also one of a small number of international trials [15] to incorporate a longer-term follow-up at six-month post intervention as a way of assessing the sustainability of any effects for a period after the intervention has concluded. This six-month post intervention phase is crucial as it is during this period that the career plan is implemented and the job-seeker independently engages in job search-related activities. Research on re-employment shows that self-regulation and effort are important in job-seeking and that individuals differ in their ability in this respect [79]. For some job-seekers, discouragement, rejection and uncertainty may make the job-seeking process more difficult [9]. Furthermore, job search activities which are non-self-determined (i.e. carried out because of pressure to do so (as in the case of conditionality) as opposed to the individuals’ own volition), have been associated with procrastination which, in turn, has been linked with increased hopelessness [80]. In addition, the relationship between job search and mental health has been shown to be negative in the short term, although there is significant research confirming the positive relationship between mental health and re-employment [81, 82], and therefore the role of job search behaviour in re-accessing the labour market. Therefore, the possible maintenance of positive wellbeing and employability during this six-month post-intervention phase could be fundamental to re-employment success.

The trial design has a number of strengths. First, the location of the trial enables access to an existent group of long-term unemployed job-seekers who are in receipt of a Job Seekers payment and who are obligated, therefore, to participate in the Pathways to Work programme/service as usual. This ensures that all potential participants are eligible, meet the inclusion criteria and expect to receive, at a minimum, the service as usual. Second, the data manager performs the randomisation, thereby reducing potential selection bias and participants are assigned thereafter to the intervention or ‘service as usual’ after baseline assessments have been completed. Participants are analysed ‘as randomised’, thereby maintaining participants in their allocated groups and further reducing any selection bias. Delivery of the intervention and ‘service as usual’ with fidelity also aims to limit participants’ likelihood to avoid some aspects of the interventions. Third, all randomised participants will be included in the analysis as per the ITT principle. As detailed earlier, an advantage of the MMRM analysis is that it allows for different numbers of measurements for each participant, and uses all available data, thus minimising attrition bias. Lastly, the researcher (initially) and the participants are blinded thereby reducing potential bias in implementation of the services and in the performance of the participants.

There are, however, also limitations to this study. First and foremost, the duration of intervention and control conditions will vary as individual needs differ. To allow for this, the extent of the intervention or control conditions will be documented in terms of the number of contact hours provided across the number of weeks of engagement with the service. These types of data could benefit the design of a model which promotes individualised approaches. Second, the NGO participating in the trial is implementing government policy, which could change at any time. The study is being conducted in a rapidly changing environment, where neither the NGO nor the researcher has the authority to reverse policy decisions. This leaves the trial vulnerable to external influences beyond our control.

Nevertheless, the trial is unique in terms of its timing and its potential contribution towards effective engagement with the long-term unemployed in Irish labour market activation. If the results of the trial show that the positive psychological intervention is superior to the ‘service as usual’ in terms of increases in employability-related outcomes, it will provide important evidence to support the further design and implementation of a more therapeutic approach to job-seeking support for long-term unemployed job-seekers. It may also provide a model of good practice that could be replicated elsewhere while also identifying key implementation ‘lessons’ for similar services in other jurisdictions. For these reasons, a mini-process evaluation will be embedded within the trial, running in parallel with the study. A small number of participants, practitioners and managers of services will be invited to participate in a one-to-one interview, in order to capture their experiences of participating in the EEPIC intervention, both in terms of its content and implementation. This process evaluation will be important in terms of supplementing and amplifying the RCT findings by adding to our understanding as to whether the intervention works, how and why it works, and for whom and under what circumstances [4].

The findings from this study will also help to inform future policy in terms of highlighting what is needed to develop an increasingly sustainable labour force.

### Trial status

The trial started in September 2016. To date, 140 participants have been randomly assigned.

## Additional files

Additional file 1: SPIRIT checklist. (DOCX 61 kb)

Additional file 2: Participant questionnaire. (PDF 511 kb)

Additional file 3: Sample participant information sheet and consent form. (PDF 338 kb)

Additional file 4: Participant update questionnaire. (PDF 292 kb)

## Abbreviations

ALMP: Active labour market policy
ARM: Activation Review Meeting
CBT: Cognitive behavioural therapy
CSO: Central Statistics Office
DSP: Department of Social Protection (renamed the DEASP- Department of Employment Affairs and Social Protection (July, 2017)
GIS: Group Information Session
Intreo: Public Employment Income Support Office
IQDA: Irish Qualitative Data Archive
ISSDA: Irish Social Sciences Data Archive
JSA: Job Seeker’s Allowance
JSB: Job Seeker’s Benefit
LMP: Labour market policy
MMRM: Mixed Model Repeated Measures
NGO: Non-governmental organisation
OECD: Organisation for Economic Co-operation and Development
PEX: Probability of Exit
PPP: Personal Progression Plan
PRSI: Pay-Related Social Insurance
PTWP: Pathways to Work Programme
RCT: Randomised controlled trial

## Footnotes

1 Unemployment rose from 4.4% in early 2008 to 15.1% in 2012 (CSO [3]).

2 Emerge was an initiative of the Mount Street Trust Employment Programme where a high support guidance intervention, based on a comprehensive profile of needs, was piloted with a sample of long-term unemployed in a disadvantaged urban area.

3 Ballymun Youth Guarantee pilot was a joint EU and Government of Ireland (Department of Social Protection) funded pilot implemented during the period 2013–2014 in the Ballymun area.
