# Supplementary material for: EEPIC - Enhancing Employability through Positive Interventions for improving Career potential: the impact of a high support career guidance intervention on the wellbeing, hopefulness, self-efficacy and employability of the long-term unemployed - a study protocol for a randomised controlled trial
Source: Trials. 2018 Feb 26;19:141. doi: 10.1186/s13063-018-2485-y (PMC5828489; doi:10.1186/s13063-018-2485-y)
Supplement: Supplementary file 2 — Participant questionnaire. (PDF 511 kb) [file 13063_2018_2485_MOESM2_ESM.pdf]

EEPIC

(**E**nhancing **E**mployability through **P**ositive **I**nterventions for improving **C**areer potential)

Participant Questionnaire

Note:
Thank you for completing the EEPIC questionnaire. Your answers will help us to improve the quality of the services we provide to you and future clients. We are interested in finding out how you are feeling about yourself and your career at this point in time.

Remember

- Please answer the following questions as honestly as you can
- Answer all the questions
- It should take 5 - 7 minutes to complete
- If you are unsure of a question / need assistance in completing the questionnaire please ask the administrator to assist you
- Your answers will give us an indication as to how you are getting on
- You may be asked to answer these questions / similar questions again in a few months time.
- Your answers are confidential. They will be entered into a database and will not be associated with your name (only linked back to you via an ID number)
- All participants in EEPIC are being asked to complete this questionnaire

Thank you very much for your help and co-operation!

Official Use only:

Date of Completion: Administrator:
ID number:

Please read this carefully:
We would like to know how you have been feeling in general, *over the past few weeks*. Please answer ALL the questions simply by circling the answer which you think most applies to you. Remember that we want to know about how you are feeling today and how you've been feeling recently, not about how you have been feeling in the past. It is important that you try to answer ALL the questions. Thank you very much for your co-operation.

HAVE YOU RECENTLY:

1 -	been able to concentrate on whatever you're doing?	Better than usual	Same as usual	Less than usual	Much less than usual
2 -	lost much sleep over worry?	Not at all	No more than usual	Rather more than usual	Much more than usual
3 -	felt that you are playing a useful part in things?	More so than usual	Same as usual	Less useful than usual	Much less useful
4 -	felt capable of making decisions about things?	More so than usual	Same as usual	Less so than usual	Much less capable
5 -	felt constantly under strain?	Not at all	No more than usual	Rather more than usual	Much more than usual
6 -	felt you couldn't overcome your difficulties?	Not at all	No more than usual	Rather more than usual	Much more than usual
7 -	been able to enjoy your normal day-to-day activities?	More so than usual	Same as usual	Less so than usual	Much less than usual
8 -	been able to face up to your problems?	More so than usual	Same as usual	Less able than usual	Much less able
9 -	been feeling unhappy and depressed?	Not at all	No more than usual	Rather more than usual	Much more than usual
10 -	been losing confidence in yourself?	Not at all	No more than usual	Rather more than usual	Much more than usual
11 -	been thinking of yourself as a worthless person?	Not at all	No more than usual	Rather more than usual	Much more than usual
12 -	been feeling reasonably happy, all things considered?	More so than usual	About same as usual	Less so than usual	Much less than usual

Below are five statements that you may agree or disagree with. Using the 1 - 7 scale below, indicate your agreement with each item by circling the appropriate number on the line beside that item. Please be open and honest in your responding.

1. In most ways my life is close to my ideal.

1	2	3	4	5	6	7
Strongly disagree	Disagree	Slightly disagree	Neither agree nor disagree	Slightly agree	Agree	Strongly agree

2. The conditions of my life are excellent.

1	2	3	4	5	6	7
Strongly disagree	Disagree	Slightly disagree	Neither agree nor disagree	Slightly agree	Agree	Strongly agree

3. I am satisfied with my life.

1	2	3	4	5	6	7
Strongly disagree	Disagree	Slightly disagree	Neither agree nor disagree	Slightly agree	Agree	Strongly agree

4. So far I have gotten the important things I want in life.

1	2	3	4	5	6	7
Strongly disagree	Disagree	Slightly disagree	Neither agree nor disagree	Slightly agree	Agree	Strongly agree

5. If I could live my life over, I would change almost nothing.

1	2	3	4	5	6	7
Strongly disagree	Disagree	Slightly disagree	Neither agree nor disagree	Slightly agree	Agree	Strongly agree

Below is a list of statements dealing with your general feelings about yourself.

If you strongly agree with the statement, circle SA.
If you agree, circle A.
If you disagree with the statement, circle D.
If you strongly disagree, circle SD.

	Strongly agree	Agree	Disagree	Strongly Disagree
1. On the whole, I am satisfied with myself.	SA	A	D	SD
2. At times, I think I am no good at all.	SA	A	D	SD
3. I feel that I have a number of good qualities.	SA	A	D	SD
4. I am able to do things as well as most other people.	SA	A	D	SD
5. I feel I do not have much to be proud of.	SA	A	D	SD
6. I certainly feel useless at times.	SA	A	D	SD
7. I feel that I'm a person of worth, at least on an equal plane with others.	SA	A	D	SD
8. I wish I could have more respect for myself.	SA	A	D	SD
9. All in all, I am inclined to feel that I am a failure.	SA	A	D	SD
10. I take a positive attitude toward myself.	SA	A	D	SD

Below are a few more similar items. Please respond to each item by ticking the box that most closely reflects how you feel (i.e. one box per row should be marked)

	**Strongly Disagree**	**Disagree**	**Neutral**	**Agree**	**Strongly Agree**
1. I tend to bounce back quickly after hard times					
2. I have a hard time making it through stressful events.					
3. It does not take me long to recover from a stressful event.					
4. It is hard for me to snap back when something bad happens					
5. I usually come through difficult times with little trouble.					
6. I tend to take a long time to get over set-backs in my life.					

Next, please take a few moments to focus on yourself and what is going on in your life at this moment. Once you have this "here and now" mindset, go ahead and answer each item according to the scale below.

For each statement, please select the answer that best describes *how you think about yourself right now* and place a tick in the box to indicate your answer.

DO be sure to read each item very carefully and answer as honestly as you can.

	1<br>*Definitely False*	2<br>*Mostly False*	3<br>*Somewhat False*	4<br>*Slightly False*	5<br>*Slightly True*	6<br>*Somewhat True*	7<br>*Mostly True*	8<br>*Definitely True*
If I should find myself in a jam, I could think of many ways to get out of it								
At the present time, I am energetically pursuing my goals								
There are lots of ways around any problem that I am facing now								
Right now I see myself as being pretty successful								
I can think of many ways to reach my current goals								
At this time, I am meeting the goals that I have set for myself								

Below are eleven statements that you may agree or disagree with. Using the 1 - 5 scale below, indicate your agreement with each item by circling the appropriate number on the line beside that item. Please be open and honest in your responding.

1. When I make plans for my career, I am confident I can make them work	*1*<br>Disagree strongly	*2*<br>Disagree slightly	*3*<br>Neutral	*4*<br>Agree slightly	*5*<br>Agree strongly

2. If I can't do a job the first time I keep trying until I can

1	2	3	4	5
Disagree strongly	Disagree slightly	Neutral	Agree slightly	Agree strongly

3. When I set important career goals for myself, I rarely achieve them

1	2	3	4	5
Disagree strongly	Disagree slightly	Neutral	Agree slightly	Agree strongly

4. I avoid facing career difficulties

1	2	3	4	5
Disagree strongly	Disagree slightly	Neutral	Agree slightly	Agree strongly

5. When I have something unpleasant to do that will help my career, I stick with it until I am finished

1	2	3	4	5
Disagree strongly	Disagree slightly	Neutral	Agree slightly	Agree strongly

6. When I decide to do something about my career, I go right to work on it

1	2	3	4	5
Disagree strongly	Disagree slightly	Neutral	Agree slightly	Agree strongly

7. When trying to learn something new on my job, I soon give up if I am not initially successful

1	2	3	4	5
Disagree strongly	Disagree slightly	Neutral	Agree slightly	Agree strongly

8. I avoid trying to learn new things that look too difficult for me

1 Disagree strongly	2 Disagree slightly	3 Neutral	4 Agree slightly	5 Agree strongly

9. I feel insecure about my ability to get where I want

1 Disagree strongly	2 Disagree slightly	3 Neutral	4 Agree slightly	5 Agree strongly

10. I rely on myself to accomplish my career goals

1 Disagree strongly	2 Disagree slightly	3 Neutral	4 Agree slightly	5 Agree strongly

11. I do not seem capable of dealing with most problems that come up in my career

1 Disagree strongly	2 Disagree slightly	3 Neutral	4 Agree slightly	5 Agree strongly

Thank You!
