# Supplementary material for: EEPIC - Enhancing Employability through Positive Interventions for improving Career potential: the impact of a high support career guidance intervention on the wellbeing, hopefulness, self-efficacy and employability of the long-term unemployed - a study protocol for a randomised controlled trial
Source: Trials. 2018 Feb 26;19:141. doi: 10.1186/s13063-018-2485-y (PMC5828489; doi:10.1186/s13063-018-2485-y)
Supplement: Supplementary file 3 — Sample participant information sheet and consent form. (PDF 338 kb) [file 13063_2018_2485_MOESM3_ESM.pdf]

Participant Information Sheet – Job seeker

Title of Research Project: **Evaluating the Effectiveness and Implementation of new employment enhancement programmes in an Irish context**

- You have been invited to participate in a research study.
- This sheet provides you with information about the study which will help you to decide if you would like to take part.
- Before you decide whether you would like to participate, it is important that you understand what the research is about.
- If any of the information provided is unclear or if you have any questions, please let me know as I would be happy to explain further or give you more information.

Details about the Researcher:

This research study is being carried out by Nuala Whelan a registered PhD Psychology student at the Department of Psychology, National University of Ireland Maynooth. Nuala is also a full time staff member at the Ballymun Job Centre, Dublin.

Purpose of the Study:

This study aims to assess the effectiveness of the employment supports you receive, most specifically the Pathways to Work programme (PTWP), which is currently delivered to job seekers through the Department of Social Protection (DSP) services and in some areas by the Local Employment Services.

This research will examine how job seekers perceive the service to be working based on their interaction with it. The impact of two types of supports will be measured with a sample of job seekers.

Why have you been asked to take part?

You have been asked to participate in this study because you are currently a jobseeker.

Do you have to take part?

- Participation is voluntary. It is your choice whether to participate or not.
- You may change your mind at any stage and withdraw from the process.
- You may be asked to participate over a period of time: at the beginning of your interaction with services and again towards the end (follow-up).

- You will be contacted in advance of the follow-up and consent will be sought. If you do not wish to continue at this time, you will be invited to complete the withdrawal slip at the end of this information sheet and return to the researcher for record purposes and all of your data will be destroyed.
- It is also important to note that you can stop and withdraw at any point in the process up to publication.

What will you have to do?

Your participation will involve some or all of the following:

- Participation in a focus group with other job seekers (approx. 1 hour duration)
- Participation in a one-to-one interview (approx. 45minutes to 1 hour duration)
- Participation in a study where you will be asked to complete a number of brief and easy-to-complete questionnaires relating to how you feel about yourself and your career. You will be asked to complete these questionnaires before your first appointment with the Ballymun Local Employment Service and again after your last appointment with the service. Each session will last approx. 40 mins[1].
- As part of this study there are two services that are being compared. We do not know as yet which of these two services is more effective. In order to test this, you will be randomly assigned to one or the other service which means that you have an equal chance of being in either. This is just like putting names into a hat and then drawing them out at random to decide who should go into which group. This is the best way of deciding which of two services works better. The two services are described briefly below.
    - Service 1: the Pathways to Work 'usual service' where participants will receive employment support services consisting of a group engagement session (approx. 30 mins), an initial one-to-one meeting (approx. 40mins) and follow up meetings every three months.
    - Service 2: a career guidance type intervention where participants will receive individualised job seeking support which includes career guidance and coaching over approx. 6-8 one hour sessions.

Will your participation in the study be kept confidential and anonymous?

- Yes - no identifying information will be included within any aspect of the study.
- You will be allocated a unique code at the point of consent (to participate) so as to anonymise the data from the outset.
- A document containing the coding key will be stored in a locked filing cabinet in the researcher's office at NUIM and will be accessed only by the researcher.

[1] To be adjusted depending on the pilot study.

What will happen to the information which you give?

- Interviews and focus groups will be audio-recorded, but no-one will be identified by name on the tape. The audio files will be kept in a password protected laptop protected by encryption software.
- Questionnaires will be completed on paper copy, with the participant's unique code as the identifier. This data will then be entered into a database on the researcher's laptop and the paper copies stored securely in the researcher's office at NUIM for a 10 year period, or until final publication.

What will happen to the results?

The results will be seen by the researcher, the supervisor and relevant examiners. The results will be presented in the published thesis and may also be presented at relevant conferences.

What are the possible disadvantages of taking part?

We do not envisage any negative consequences for participants in taking part in this important study. There is no conditionality related to this study so no penalties for non –participation will apply.

Any further queries?

If you need any further information, please feel free contact me.

Researcher: Nuala Whelan, B.A. (Hons.) Psychology, M.Sc. Industrial Psychology

Department of Psychology, NUIM, Maynooth, Co. Kildare

Tel: (01) 7086734

Nuala.whelan.2014@mumail.ie

The research supervisors are Prof. Sinead McGilloway and Dr. Mary Murphy who can be contacted as below:

Supervisors: Prof. Sinead McGilloway, Department of Psychology, NUIM, Maynooth, Co.Kildare

Tel: (01) 7086052/7084765

Sinead.Mcgilloway@mumail.ie

Dr. Mary P. Murphy, Department of Sociology, NUIM, Maynooth, Co. Kildare

Tel: (01) 7086556

Mary.p.murphy@mumail.ie

If during your participation in this study you feel the information and guidelines that you were given have been neglected or disregarded in any way, or if you are unhappy about the process, please contact the Secretary of the National University of Ireland Maynooth Ethics Committee at research.ethics@nuim.ie or +353 (0)1 708 6019. Please be assured that your concerns will be dealt with in a sensitive manner.

If you would like to **withdraw** from this study at any point, please sign below and return this form immediately to me at:

Nuala Whelan

Department of Psychology, NUI Maynooth, Maynooth, Co. Kildare

Signed:

Dated:

Informed Consent Form

Research Project entitled: Evaluating the Effectiveness and Implementation of new employment enhancement programmes in an Irish context

Please read and sign this form if you would like to participate in this study

<u>I understand the following:</u>

- This research study will be carried out by Nuala Whelan, B.A., MSc., a registered PhD Psychology student at the Department of Psychology, National University of Ireland Maynooth, Co. Kildare. Nuala is also a full time staff member at the Ballymun Job Centre, Dublin.
- Participation is voluntary. It is my choice whether to participate or not. I may change my mind at any stage and withdraw from the process. I may be required to participate on more than one occasion as a follow-up questionnaire may be conducted. I will be contacted in advance of the follow-up and consent will be sought. If I do not wish to continue at any time, I will be requested to complete the withdrawal slip. This will be returned to the researcher for record purposes. Should this occur, all of my personal data will be destroyed.

- My participation in the study will be kept confidential and anonymous. No identifying information will be included within the transcripts/questionnaires nor will any information be included in the final write-up of the research. Any extracts from what I say that are quoted in the research report, will be entirely anonymous. The identities of participants/ interviewees will be concealed in all documents resulting from the research ensuring anonymity.
- I understand that I may be asked to be involved in some or all of the following:
    - a focus group with other job seekers/stakeholders (approx. 1 hour duration)
    - an interview with the researcher (approx. 1 hour duration)
    - a study where I will be asked to complete several questionnaires relating to how I feel across a number of areas.
    - (For Job seekers only) I understand that there are two services and I will be randomly assigned into one or the other, with an equal chance of being in either. The services have been explained to me
- All participants will be allocated a code at the point of consent (to participate) so as to anonymise the data from the outset. A document containing the coding key will be stored in a locked filing cabinet in the researcher's office at NUIM and accessible only by the researcher. All coded data will be stored on the researcher's laptop and protected by encryption software.
- All Interviews and focus groups will be audio-recorded (with my consent), but no-one will be identified by name on the tape. The audio files will be kept in a password protected computer protected by encryption software. Questionnaires will be completed on paper copy, with my unique code as the identifier. These data will then be entered into a database on the researcher's computer and the paper copies stored securely in the researcher's office at NUIM until the point of final publication/10years. All of the information recorded is confidential.
- The results will be seen by the researcher, the supervisor and relevant examiners. The results will be presented in a thesis/report and may also be presented at relevant conferences and published in academic journals and, where applicable, in other outlets.
- There are no anticipated risks or negative consequences envisaged for participants taking part. There is no conditionality related to this study, no penalties for non–participation apply. The researcher is responsible for adhering to the ethical guidelines of the Psychological Society of Ireland.
- I have been provided with an information sheet related to this research project.
- I will receive a copy of this signed consent form for my own records.
- I may contact the researcher at point if I have any questions or concerns regarding my participation in this study.

Researcher: Nuala Whelan, B.A. (Hons.) Psychology, M.Sc. Industrial Psychology

Department of Psychology, NUIM, Maynooth, Co. Kildare

Tel: (01) 7086734

Nuala.whelan.2014@mumail.ie

Supervisors: Prof. Sinead McGilloway, Department of Psychology, NUIM, Maynooth, Co.Kildare

Tel: (01) 7086052

Sinead.Mcgilloway@mumail.ie

Dr. Mary P. Murphy, Department of Sociology, NUIM, Maynooth, Co. Kildare

Tel: (01) 7086556

Mary.p.murphy@mumail.ie

I have read and understand the information provided on the Information Sheet and the Consent form and agree to voluntarily participate in this research.

Signed: ______________________________

Date: ________________________________
