# Supplementary material for: EEPIC - Enhancing Employability through Positive Interventions for improving Career potential: the impact of a high support career guidance intervention on the wellbeing, hopefulness, self-efficacy and employability of the long-term unemployed - a study protocol for a randomised controlled trial
Source: Trials. 2018 Feb 26;19:141. doi: 10.1186/s13063-018-2485-y (PMC5828489; doi:10.1186/s13063-018-2485-y)
Supplement: Supplementary file 4 — Participant update questionnaire. (PDF 292 kb) [file 13063_2018_2485_MOESM4_ESM.pdf]

EEPIC

(**E**nhancing **E**mployability through **P**ositive **I**nterventions for increasing **C**areer potential)

Participant Update Questionnaire

EMPLOYMENT

1. **Are you currently employed ○ or unemployed ○?**
    a. **Full time employed**
    b. **Part time employed**
    c. **Community Employment Scheme**
    d. **Other**

2. **What is your job title: _______________________________________**

3. **All in all, how satisfied would you say you are with your new job?** Circle your answer:

   Not at all satisfied   Fairly Satisfied   Satisfied   Very satisfied

3. **How likely is it that you will actively look for another job in the next year?**

   1 2 3 4 5 6 7

   Not at all likely   Very likely

4. **How satisfied are you with your level of earnings?**

   1 2 3 4 5

   Dissatisfied   Very Satisfied

EDUCATION or TRAINING

5. **Are you currently registered on an education or training course relevant to your career plan?**
   - ○ Yes
   - ○ Waiting to start
   - ○ Already completed this training
   - ○ No, I am not participating in education or training

   Training Course Name: _______________________________________

Thank you!
